# Behind the Screens : Exploring the phenomenon of Binge-Watching behaviour among Tunisian Adolescents

**DOI:** 10.1192/j.eurpsy.2025.950

**Published:** 2025-08-26

**Authors:** R. Ayoub, F. Jemil, N. Fleh, I. Gouia, H. Ben Abid, A. Guedria

**Affiliations:** 1 Child and Adolescent Psychiatry Departement, Fattouma Bourguiba Hospital; 2 University of Monastir, Monastir, Tunisia

## Abstract

**Introduction:**

In recent years, the rise of streaming platforms has given adolescents unprecedented access to vast libraries of series and films, often leading to hours of continuous viewing, a phenomenon known as binge watching. This emerging trend is reshaping how adolescents engage with media, often affecting their social behaviors, routines, and overall well-being. Understanding these factors is crucial for developing preventive interventions.

**Objectives:**

This study aims to explore binge-watching behavior among Tunisian adolescents and the key factors influencing this trend.

**Methods:**

We conducted a descriptive and analytical cross-sectional study among Tunisian adolescents aged between 12 to 18 years old. Data were collected using an online questionnaire spread throughout social media (Facebook), using the Google Forms® platform in September 2024. We evaluated the epidemiological and social characteristics of the participants, as well as the binge watching behaviour, using Binge-Watching Addiction Questionnaire (BWAQ).

**Results:**

Eighty-two adolescents participated in our study, with a mean age of 16.02 ± 1.65 years and a sex ratio of 0.82. Our resultas showed that 86.5% of our population lived in urban areas, 13.4% in rural areas, and 18.3% did not live with both parents. Our results showed that 15.9% of our population reported low grades, 23.2% had repeated a grade, and 27.7% had issues with teachers or administration. Additionally, 21.5% had experienced bullying, 30.5% faced domestic violence, and 31.7% reported conflicts with parents. Regarding binge-watching, 41.5% of participants engaged in it more than once per week, 43.1% spent over two hours daily on school days, and 29.3% watched more than five episodes in one sitting. The most preferred content included drama series (56.1%), movies (24.4%), and sports shows (19.6%).The mean score on the Binge-Watching Addiction Questionnaire (BWAQ) was 39.43, with 27.7% showing moderate and 7.7% showing highly problematic binge-watching, with no sex difference. Highly problematic binge-watching was significantly associated with conflicts with parents (p = 0.01), peers (p = 0.02), school staff (p = 0.42), and poor academic performance, including grade repetition (p < 0.01).

**Conclusions:**

These results highlight the potential negative impact of excessive media consumption on adolescents’ social interactions and and academic performance, underscoring the need to implement targeted interventions that not only raise awareness about the risks of excessive media consumption but also provide practical strategies to help adolescents manage their screen time.

**Disclosure of Interest:**

None Declared

